# Breast cancer incidence and mammography screening among resettlers in Germany

**DOI:** 10.1186/s12889-020-08534-7

**Published:** 2020-03-30

**Authors:** Simone Kaucher, Laura Khil, Hiltraud Kajüter, Heiko Becher, Maren Reder, Petra Kolip, Jacob Spallek, Volker Winkler, Eva-Maria Berens

**Affiliations:** 1 Unit of Epidemiology & Biostatistics, Institute of Global Health, University Hospital Heidelberg, Im Neuenheimer Feld 324, 69120 Heidelberg, Germany; 2 Federal Cancer Registry of North Rhine-Westphalia, Bochum, Germany; 3 Institute of Medical Biometry and Epidemiology, University Medical Center Hamburg-Eppendorf, Hamburg, Germany; 4 Institute of Psychology, University of Hildesheim, Hildesheim, Germany; 5 Department of Prevention and Health Promotion, Bielefeld School of Public Health, Bielefeld University, Bielefeld, Germany; 6 Department of Public Health, Brandenburg University of Technology, Senftenberg, Germany; 7 Department of Health Services Research and Nursing Science, Bielefeld School of Public Health, Bielefeld University, Bielefeld, Germany

**Keywords:** Resettlers, Migrants, Breast cancer, Stage at diagnosis, Mammography screening programme, Participation

## Abstract

**Background:**

European studies showed that women with a migration background are less likely to participate in mammography screenings than autochthonous women. However, the participation in the German mammography screening programme (MSP) among ethnic German migrants from countries of the former Soviet Union (called resettlers) is unclear so far. The aim of this study was to identify possible differences regarding MSP participation between resettlers from the FSU and the general German population.

**Methods:**

Data from two independent, complementary studies from North Rhine-Westphalia, Germany (a retrospective cohort study 1994–2013; a cross-sectional study 2013/14) were used for comparisons between resettlers and the general population: Odds Ratios (ORs) for MSP participation utilizing the cross-sectional data and time trends of breast cancer incidence rates as well as Chi-Square tests for breast cancer stages utilizing the cohort data.

**Results:**

Resettlers showed higher Odds to participate in the MSP than the general population (OR 2.42, 95% CI 1.08–5.42). Among resettlers, a large increase in incidence rates was observed during the MSP implementation (2005–2009), resulting in stable and comparable incidence rates after the implementation. Furthermore, pre-MSP implementation, the proportion of advanced breast cancer stages was higher among resettlers than in the German population, post-MSP implementation the proportion was comparable.

**Conclusions:**

MSP participating seems surprisingly high among resettlers. An explanation for the increased willingness to participate might be the structured invitation procedure of the MSP. However, the exact reasons remain unclear and future research is needed to confirm this hypothesis and rule out the possibility of selection bias in the cross-sectional study.

## Background

With around 69,000 new cases in 2014, breast cancer is the most common cancer among women in Germany [1]. European studies have shown that non-western migrants are less likely to develop breast cancer than individuals in autochthonous populations [2]. Such differences in breast cancer development could be due to varying lifestyle and reproductive factors between the populations [2, 3]. Additionally, lower participation in mammography screening has been shown among migrant women, which could at least partially explain the lower breast cancer incidence in these women [4–7]. In addition, studies from the US found that migrant women with increasing length of stay in the country of destination showed higher risks of breast cancer but also participated more frequently in mammography screening than migrants with a shorter length of stay [3, 8].

In Germany, the population-based mammography screening programme (MSP) was introduced in 2005 and was fully implemented nationwide by 2009. Women between 50 and 69 years of age are invited biennially to participate in the MSP. The aim is to diagnose breast cancer at an early stage, and thus, to improve medical treatment and reduce breast cancer mortality [9]. Within the last decade, there has been a significant increase in breast cancer incidence in Germany, which is likely the result of the introduced MSP [10].

The European guidelines for quality assurance in breast cancer screening and diagnosis define the aim to have at least 70–75% of invited women participate in the MSP to assure effectiveness of the programme [9]. However, a previous study showed that only nine of 26 European programmes achieved this level [11]. In Germany, the proportion of participation is largely stable at around 55% since the introduction of the MSP [12–15].

Resettlers (in German: *(Spät-) Aussiedler*) are ethnic German migrants, whose ancestors emigrated to Russia in the 18th and 19th centuries. After the collapse of the Soviet Union, a huge number of ethnic Germans from countries of the former Soviet Union (FSU) migrated back into Germany. Resettlers are identified as a unique migrant population, as upon arrival in Germany they receive both the German citizenship, and unrestricted access to German social and health care systems [16]. In 2011, approximately 3.2 million resettlers lived in Germany, making resettlers one of the largest migrant groups in Germany. In 2018, about 39% of resettlers included in the micro-census were between age 50 and 69 compared to 29% of the general German population. However, the micro-census data does not distinguish between resettlers coming from Romania or Poland and those coming from the FSU [17].

Previous studies showed lower rates of breast cancer incidence and mortality; but a higher proportion of advanced breast cancer stages among resettlers compared to the general German population [18, 19]. In addition, it was shown that the incidence ratio of breast cancer between resettlers and the general population converged over time [19]. Explanations pertaining to rising breast cancer incidence among resettlers have not yet been determined, and data regarding participation levels in MSP among resettlers were not available so far.

The aim of this study was to identify possible differences regarding MSP participation between resettlers from the FSU and the general German population with the help of two independent, complementary studies. Therefore, this study aims to address the following three comparisons between resettlers and the general German population: (i) MSP participation, (ii) time-trends of age-specific breast cancer incidence, and (iii) distribution of breast cancer stages at diagnosis.

## Methods

### Study populations

The present work used data obtained from two different studies conducted in the federal state of North Rhine-Westphalia (NRW), Germany. The InEMa study (Informierte Entscheidung zur Teilnahme am Mammographie-Screening-Programm, *N* = 4828 [20]) is a cross-sectional study and data were used to observe MSP participation among resettlers. Within this study, questionnaires were sent to a random sample of women residing in the area of Westfalen-Lippe (sub-area of NRW) who had their 50th birthday between October 2013 and July 2014. The sample was randomly chosen by a computer algorithm from a database of the target populations’ addresses retrieved from local registration offices within the study area. Based on the results of previous sample size calculations, it was sufficient to randomly select and contact 56% (17,349 women) of women in the target population rather than contacting all women in the database. Women were sent two questionnaires: one very soon after their 50th birthday (t1), when women in Germany are receiving their first invitation to participate in the MSP, and another one three months later (t2). The t1 questionnaire was used to collect sociodemographic data, as well as data regarding the core elements of an informed decision (participation, knowledge and attitude towards MSP) [21]. These core elements of informed decision were measured also after the women made their first decision whether or not to participate in the MSP (t2). With the information regarding the country of origin and the nationality (German, German due to resettler status, not German), resettlers from the FSU could be identified [20]. The analyses excluded all women with a breast cancer diagnosis earlier in life, because they receive mammograms as part of their follow-up visits and are not the primary target group of the MSP. In addition, women were excluded when participation status at the MSP was unclear, for example due to missing information.

The AMIN study (Aussiedler in Münster - Incidence Cohort Study, *N* = 32,972 [19]) is a retrospective, register-based cohort study that provides information on breast cancer incidence among resettlers from the FSU and the general German population. The study was conducted in the administrative district (AD) of Münster in cooperation with the federal cancer registry of NRW. The cohort includes a sample of resettlers who immigrated to the AD Münster between 1990 and 2001 and were identified with the help of local registration offices. Person-years were estimated using a validated procedure for cohort studies with an incomplete follow-up [22]. Incidence data as well as population figures of the general population in the AD Münster were available from the federal cancer registry of NRW.

Even though this study compared results from two different study populations, the same target population was investigated. Table 1 is summarising the study characteristics from the InEMa and the AMIN study.

**Table 1:** Overview of study characteristics and inclusion criteria from both studies

	InEMa cross-sectional study	AMIN cohort study
Inclusion criteria:		
Place of residence / study region	Region of Westfalen-Lippe (sub-area of North Rhine-Westphalia)	Münster (Westfalen) (administrative district in North Rhine-Westphalia)
Survey period	2013/2014	1994–2013
Further inclusion criteria	No previous breast cancer diagnosis	No previous breast cancer diagnosis
	50 years of age (eligible for MSP for the first time)	No age restriction
Resettler definition:		
	Migrated from countries of the former Soviet Union	Migrated between 1990 and 2001 (restriction to resettlers from countries of the former Soviet Union)
	German nationality	German nationality
Variables of interest:		
	• Participation in the mammography screening programme • Possible determinants and confounders • Resettler women vs. other study participants (German women and women with migration background)	• Breast cancer incidence (C50 diagnoses) • Stage at breast cancer diagnosis (TNM-based) • Resettler women (from cohort) vs. general population of Münster (including German women and women with migration background)

### Variables

In the InEMa study, participation in mammography screening was recorded in the t2 questionnaire and participants were asked whether the mammogram was performed within the MSP or within an opportunistic screening by a gynaecologist or a radiologist. In some cases, women reported already in the t1 questionnaire that they had participated in the MSP. These women were included in the analyses of this manuscript, even though they did not send back the t2 questionnaire. Additionally, further variables were recorded, which might be possible determinants of MSP participation: educational level (low / medium: no educational qualification, less than 12 years at school or an equivalent degree (in German: *Haupt- oder Realschulabschluss*); high: at least 12 years at school or an equivalent degree (in German: *(Fach-) Hochschulreife / Abitur)*), invitation status (invitation to MSP already received *yes* vs. *no*), length of stay in Germany (*since birth, since ... years*) and the language that is mainly spoken at home.

The AMIN study provides information on all cancer diagnoses from 1994 to 2013 of the population of the AD Münster. The analyses were limited to invasive breast cancer diagnoses among women (ICD-10: C50). The cancer diagnoses of resettlers from the AMIN study were identified in the federal cancer registry of NRW using a pseudonymised record-linkage procedure [23, 24]. In addition to the resettler status, the dataset contained information about the age at diagnosis, date of diagnosis and TNM status (T: tumour size, N: lymph node involvement, M: metastases). The tumour stage was defined using the Union for International Cancer Control (UICC) classification system [25]. Therefore, tumours that were smaller than 20 mm (T1) with either no or low lymph node involvement (N0 or N1mic), and in which there were no distant metastases (M0) were classified as UICC stage I (local). All larger tumours (T2-T4), tumours with lymph node involvement (N + (with the exception of N1mic)), and tumours with distant metastases (M +) were classified as UICC stage II + (advanced). Tumours with neoadjuvant therapy were classified as UICC stage 0 [25].

### Analyses

Differences in MSP participation were assessed by logistic regression, calculating univariate Odds Ratios (ORs) and adjusted ORs with 95% confidence intervals (95% CI). The multivariate model included all variables that were significant in the univariate models (*p*-value < 0.05).

For the period 1994 to 2013, age-specific and age-standardised breast cancer incidence rates (ASRs) based on the old European Standard Population were calculated for female resettlers in the AMIN cohort and the female population of Münster [26]. The rates of resettlers were calculated for 3 years combined. The age-specific rates were combined for three different age groups: (a) < 50 years, (b) between 50 and 69 years, and (c) ≥ 70 years. Differences in breast cancer stages between resettlers and the general German population were tested for significance using Chi-Square tests. Two different periods (pre- and post-MSP implementation) were considered: 1994–2006 and 2007–2013. The cut point 2006 was used even though the MSP implementation began in October 2005, because fewer than 50% of eligible women were invited until 2006 [13]. All analyses were performed using SAS version 9.4.

## Results

In the InEMa study, 17,349 women were invited to participate in the study, of which 5847 women responded (response: 33.7%). After 3 months, these women were asked to complete a further questionnaire, which was completed by 4964 women (response: 84.9%). Women with a previous breast cancer diagnosis (*N* = 167) and women who did not return the second questionnaire were excluded from the analyses. However, 31 women indicated in the first questionnaire that they had already participated in the MSP and thus, were directly included in the analyses (even in the absence of the second questionnaire). Finally, 4828 women from the InEMa study were included in the analyses (69 of them were resettlers). The AMIN study identified a total of 16,939 female resettlers (accumulating a total of 249,250 person-years) in the AD Münster and included them in the cohort. On average, they were 30.3 years of age on arrival.

Further, the majority of resettlers migrated from Russia and Kazakhstan to Germany from 1990 onwards and were living in the north-eastern part of NRW at the time of the study. Table 2 presents the data from the InEMa study regarding the country of origin, the immigration periods and the language that is mostly spoken at home, as well as the educational level and whether or not the women had received the invitation to MSP. It was found that the majority of resettlers of the InEMa study had a low to medium educational level (62.3%) and most frequently spoke German and Russian in combination (53.6%). In comparison to the other study participants, resettlers participated more often in MSP (84.1% vs. 73.6%, *p*-value: 0.02).

**Table 2:** Study characteristics of the InEMa study, separated by resettlers and other study participants and MSP participation

	resettlers (***N*** = 69)			Other study participants (***N*** = 4759)		
	Had participatedin MSP^a^	Had not participated in MSP^a^	Total^**c**^	Had participated in MSP^b^	Had not participated in MSP^b^	Total^**c**^
**Participation in the mammography screening programme (MSP)**	58 (84.1%)	8 (11.6%)	**66 (95.7%)**	3504 (73.6%)	1122 (23.6%)	**4626 (97.2%)**
**Country of origin**						
Germany	–	–	**–**	3237 (92.4%)	1062 (94.7%)	**4414 (92.8%)**
Russia	33 (56.9%)	4 (50.0%)	**39 (56.5%)**	17 (0.5%)	1 (0.1%)	**18 (0.4%)**
Kazakhstan	19 (32.8%)	4 (50.0%)	**24 (34.8%)**	5 (0.1%)	3 (0.3%)	**8 (0.2%)**
other countries of origin	6 (10.3%)	–	**6 (8.7%)**	245 (7.0%)	56 (5.0%)	**319 (6.7%)**
missing	–	–	**–**	–	–	**–**
**Immigration period**						
born in Germany	–	–	**–**	3237 (92.4%)	1062 (94.7%)	**4414 (92.8%)**
before 1990	10 (17.3%)	1 (12.5%)	**11 (15.9%)**	178 (5.1%)	37 (3.3%)	**228 (4.8%)**
1990 or later	48 (82.8%)	7 (87.5%)	**58 (84.1%)**	87 (2.5%)	23 (2.0%)	**114 (2.4%)**
missing	–	–	**–**	2 (0.1%)	–	**3 (0.1%)**
**Language that is mainly spoken at home**						
German	22 (37.9%)	3 (37.5%)	**25 (36.2%)**	3285 (93.8%)	1081 (96.3%)	**4479 (94.1%)**
Russian	6 (10.4%)	1 (12.5%)	**7 (10.1%)**	4 (0.1%)	1 (0.1%)	**4 (0.1%)**
other languages	–	–	**–**	46 (1.3%)	12 (1.1%)	**66 (1.4%)**
German in combination with a different language	30 (51.7%)	4 (50.0%)	**37 (53.6%)**	147 (4.2%)	25 (2.2%)	**181 (3.8%)**
missing	–	–	**–**	22 (2.0%)	4 (0.4%)	**28 (0.6%)**
**Educational level**						
low/medium	39 (67.2%)	6 (75.0%)	**45 (68.2%)**	2186 (62.4%)	640 (57.0%)	**2826 (61.1%)**
high	15 (25.9%)	2 (25.0%)	**17 (25.8%)**	1288 (36.8%)	473 (42.2%)	**1761 (38.1%)**
missing	4 (6.9%)	–	**4 (6.1%)**	30 (0.9%)	9 (0.8%)	**39 (0.8%)**
**Already received an invitation to MSP**						
yes	47 (81.0%)	5 (62.5%)	**52 (78.8%)**	2946 (84.1%)	643 (57.3%)	**3589 (77.6%)**
no	11 (19.0%)	2 (25.0%)	**13 (19.7%)**	541 (15.4%)	467 (41.6%)	**1008 (21.8%)**
missing	–	1 (12.5%)	**1 (1.5%)**	17 (0.5%)	12 (1.1%)	**29 (0.6%)**

Legend: ^a^missings: 3 missings among resettlers, ^b^133 missings among other participants, ^c^the sum includes the missings from participation (except the first variable)

### MSP participation

Using logistic regression, ORs for participation in the MSP between resettlers and the other study participants (women with and without migration background) were calculated, adjusting for the educational level and the invitation status. It was found that resettlers participated more frequently in MSP than the other study participants (OR 2.42, 95% CI 1.08–5.42), regardless of their educational level and whether or not they had received the invitation to MSP. Results showed that women with a lower or medium educational level and who already received the invitation to participate in the MSP had higher odds to participate in the MSP compared to women with a higher educational level and to women who did not receive the invitation for MSP participation yet. Only minor differences were observed between crude and adjusted ORs (see Table 3). In a sensitivity analysis, we excluded women with a migration background from the reference group (*N* = 345) and thus, compared resettler women with German women, however, the results differed only slightly from the previously reported results (OR 2.48, 95% CI 1.11–5.57).

**Table 3:** Crude and adjusted Odds Ratios (ORs) with 95% confidence intervals (95%CI) and *p*-values from logistic regression for MSP participation

	Crude ORs (95%CI)	*p*-value	Adjusted ORs^a^ (95%CI)	*p*-value
**Resettler status**				
yes vs. no	2.32 (1.11–4.88)	0.026	2.42 (1.08–5.42)	0.033
**Educational level**		0.001		0.001
low/medium vs. high	1.26 (1.10–1.44)		1.27 (1.10–1.46)	
**Already received an invitation to MSP**				
yes vs. no	3.92 (3.38–4.58)	< 0.001	3.96 (3.40–4.60)	< 0.001

Legend: ^a^the model of the adjusted ORs contained the three variables as independent variables: resettler status, educational level and invitation status. The reported ORs are the effect size of the respective variable, adjusted for the other variables

### Incidence time-trend analyses

**Fig. 1:**
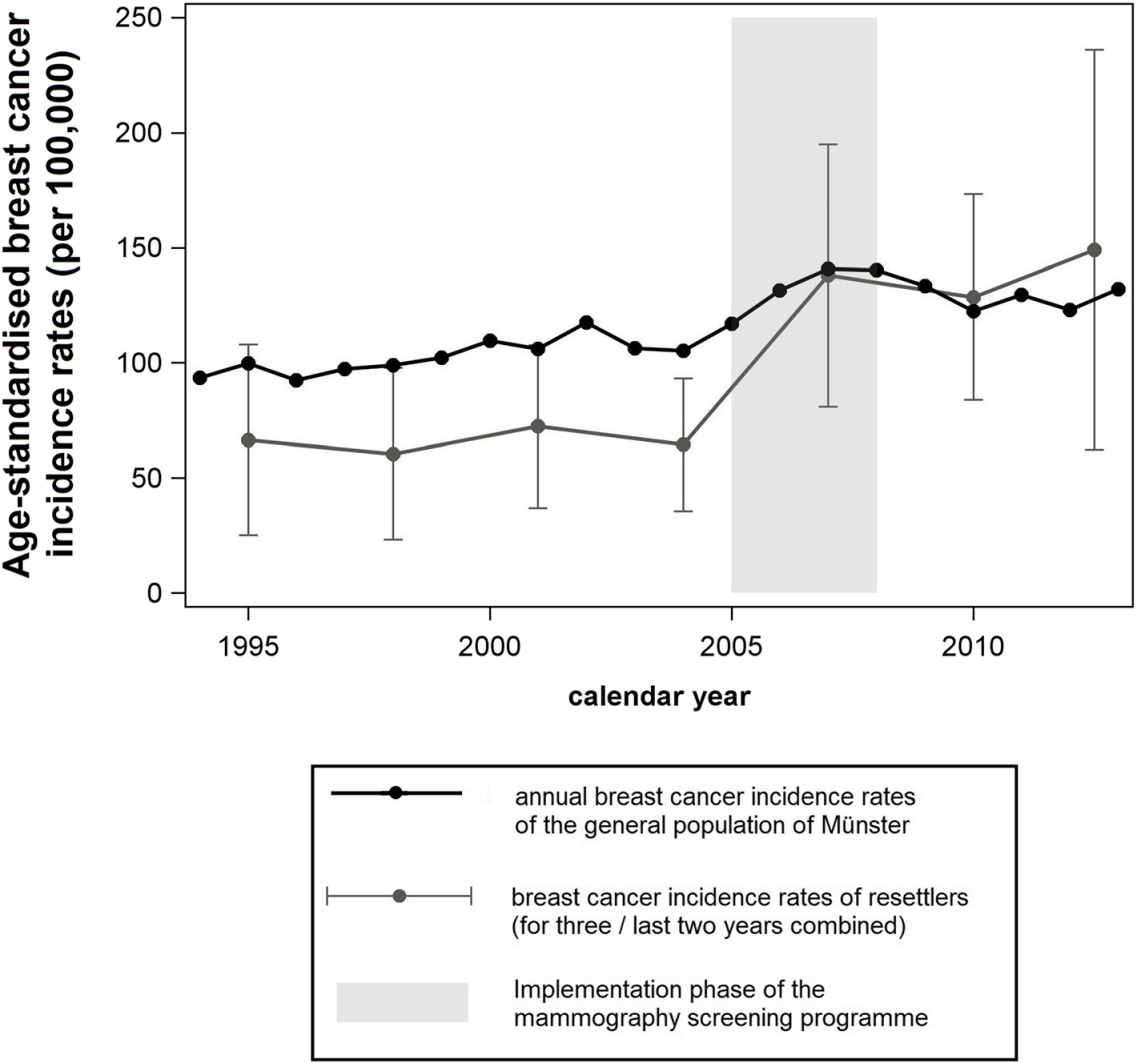
Age-standardised breast cancer incidence rates, separated for resettlers and the Münster population (1994–2013, AMIN study)

A total of 199 cases of invasive breast cancer were diagnosed among resettlers of the AMIN cohort, of which 154 (77.4%) were between 50 and 69 years of age. Figure 1 shows ASRs for all age groups.

Prior to the year 2005 (when the MSP was introduced), ASRs were stable for both groups, but lower for resettlers than in the general German population. During the implementation phase of the MSP, a strong increase in incidence rates was observed in both groups, and incidence rates among resettlers rose to a level comparable to that in the general German population. After the MSP was implemented nationwide, the incidence rates in both groups remained roughly stable.

**Fig. 2:**
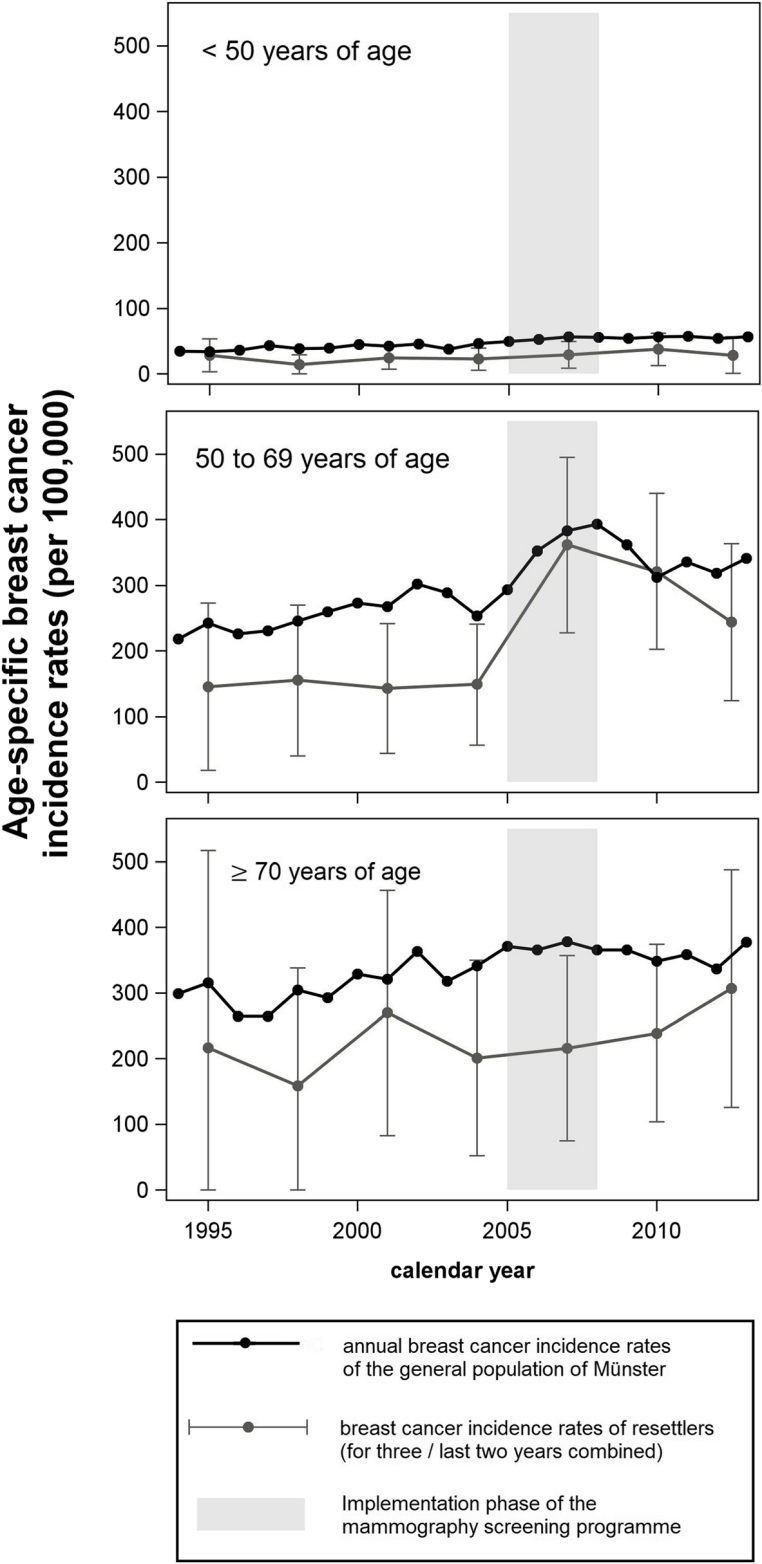
Age-specific breast cancer incidence rates, separated by resettlers and the Münster population (1994–2013, AMIN study)

Age-specific incidence rates of breast cancer, separated by three age groups can be found in Fig. 2. It shows that the observations in Fig. 1 may be explained mainly by the breast cancer incidence of women eligible to participate in the MSP. The incidence rates in under-50-year-old women remained almost unchanged. The incidence rates in over-70-year-old women showed no substantial increase during the MSP implementation phase but after 2009, the breast cancer incidence rates of resettlers and the general German population approached values similar to one another.

### Breast cancer stage analyses

Table 4 presents the distribution of UICC stages for resettlers and the general German population, separated for two time periods (1994–2006 and 2007–2013).

**Table 4:** Distribution of breast cancer stages, separated by resettlers and the Münster population (AMIN study)

UICC-stage	pre-MSP implementation: 1994–2006			post-MSP implementation: 2007–2013		
	resettlers N (%)	Münster population N (%)	Chi-Square *p*-value	resettlers N (%)	Münster population N (%)	Chi-Square *p*-value
**stage 0**	0 (0%)	23 (0.1%)	0.03	2 (1.8%)	99 (0.6%)	0.19
**UICC I (local)**	16 (18%)	4809 (21%)		38 (34.6%)	5461 (32.9%)	
**UICC II + (advanced)**	61 (68.5%)	12,252 (53.5%)		58 (52.7%)	8330 (50.2%)	
**unknown stage**	12 (13.5%)	5819 (25.4%)		12 (10.9%)	2693 (16.2%)	
Sum of diagnoses	89	22,903		110	16,583	

During the time period of 1994–2006, the proportion of advanced breast cancer stages (UICC II +) was higher in resettlers than in the general German population (68.5% vs. 53.5%). Additionally, an unknown stage was reported less frequently in resettlers. During the time period of 2007–2013, the proportion of an advanced breast cancer stage (UICC II +) of the two groups have converged to similar values and were comparable. The difference in the unknown stage was reduced, but resettlers still showed a lower proportion of unknown stages than women in the general German population. Looking only at women of the eligible age range for MSP (50 to 69 years), a similar distribution of proportions of breast cancer stages between resettlers and the general German population was observed over the two periods (data not shown).

## Discussion

The results of the InEMa study showed higher odds of MSP participation among resettlers compared to women of the general German population. Results from the AMIN study supported this finding, as a large increase in breast cancer diagnoses during the implementation phase of the MSP was observed among resettlers and the proportion of advanced breast cancer diagnoses has decreased between the two periods “1994 to 2006” and “2007 to 2013”. High participation in MSP leads to more diagnoses with earlier stages at diagnosis [27].

The findings from both studies suggest that resettler women have a surprisingly high MSP participation. The facts that these women have lived for about two decades in Germany and are able to communicate well in German might be possible reasons for the high participation of resettler women in the MSP. An ongoing process of acculturation can also increase participation in early detection measures, as studies from the US have previously observed [3, 8].

Another reason could be that resettlers are participating less in opportunistic screening, which could explain the difference in incidence rates before the MSP was implemented. This was also observed in a previous analysis of the InEMa data [28], however, in this analysis, ethnic German migrants who immigrated from Poland, Romania and countries of the FSU were considered as one homogenous group, which is different in our current analysis. Unfortunately, gynaecologists are not reporting opportunistic screening uptake to the cancer registries in Germany, so no data are available to answer this question.

In contrast, Aparicio and colleagues found that resettlers were less likely to participate in cancer screenings than the German population [29]. However, their analysis defined resettlers as ethnic Germans coming from Poland, Romania and countries of the FSU, whereas we excluded resettlers from Poland and Romania from our analyses, since we think these two groups should be investigated separately. Furthermore, the analysis looked at participation in general cancer screenings without considering the population-based MSP, which is structurally different from other early detection measures. It seems that the MSP has fewer barriers for resettlers than other early detection measures. A possible explanation might be that the structured invitation procedure of the MSP leads to an increased willingness to participate among resettlers. However, the exact reasons remain unclear and cannot be determined from the data sources we have used.

### Strengths and limitations

When comparing the immigration periods and the countries of origin of the InEMa and AMIN study, the two study populations mostly immigrated after 1990 and came primarily from the Russian Federation and Kazakhstan. Therefore, both studies reflect the expected immigration pattern of resettlers coming to Germany since the early 1990s. As shown in Table 1, both study populations are residing in the north-western part of the federal state NRW. While the results of the AMIN study cover the time period 1994–2013, the results of the InEMa study reflect the participation behaviour of 50-year-old women in 2013 and 2014. The AMIN cohort is a representative sample of resettlers from the FSU, as resettlers were quasi-randomly assigned to their first place of residence (using the *Königsteiner Schlüssel*), where they had to live for at least 2 or 3 years [16]. Possible name changes of the cohort were considered by using a name thesaurus.

In this study, we used data from two independent studies, which means that we investigated two different study populations and the comparison of the results should be treated with caution. It is to be noted that the InEMa questionnaires were distributed in the German and Turkish language. Hence, it is possible that a small proportion of women were not able to read the material as some resettlers speak only Russian at home (see Table 2). As it seems reasonable that people with poor language skills are less likely to participate in the MSP [8], it is possible that we have overestimated the association between MSP participation and resettler status. Additionally, it is possible that women with a positive attitude towards the MSP were more likely to have participated in the InEMa study. This is suggested by the high proportion of study participants who have participated in the MSP (about 80% InEMa study vs. 55% Germany-wide). Therefore, the results from the InEMa study could also be explained by selection bias, which we cannot rule out.

It should also be noted that the number of resettlers participating in the study was low, resulting in inaccurate estimates with wide confidence intervals. Previous surveys among resettlers in Germany showed a relatively low response as for example 36% [30]. An analysis of response among individuals with foreign background in Germany by Winkler and colleagues showed that study participation among resettlers is only slightly lower compared to Germans [31]. Unfortunately, it is not possible to calculate the response rate of resettlers in the InEMa study. But given the small proportion of resettlers among the overall study population, the interpretation of the results is limited.

When comparing the cancer stages between the two time periods (“1994 to 2006” vs. “2007 to 2013”), it needs to be considered that improved diagnostics may also have led to a decrease of advanced cancer stages. Furthermore, the number of 50 to 69-year old resettler women with a breast cancer diagnosis was very low in the AMIN cohort, thus, the results from the analysis of the cancer stages should be treated with caution. In the AMIN cohort, it was not possible to perform a mortality follow-up, therefore, person-years of the cohort were estimated [22]. However, sensitivity analyses showed negligible differences in the results. A detailed discussion of the strengths and weaknesses of the AMIN study has been published elsewhere [19].

It needs to be emphasised that resettlers are a specific migrant group in Germany. They are ethnic German migrants who were invited by the German government, they received German citizenship at immigration and therefore, full access to the German social and health systems [16]. In contrast to resettlers in Germany, other non-western migrants in Denmark were found to have a considerably lower willingness to participate in the MSP [6]. Therefore, further research is needed to identify the specific factors explaining the good MSP participation among resettlers which may help to examine whether we could derive our findings to other migrant groups. Furthermore, it should be investigated how resettlers are participating in other screening programmes (such as colorectal cancer screening, skin cancer screening, etc.). Results from the NAKO study, a large prospective cohort study in Germany investigating about 200,000 representative study participants, could be useful for this investigation [32].

## Conclusions

Our findings indicate a surprisingly high MSP participation among resettler women in Germany. Possibly this is due to the invitation procedure itself, however, the exact reasons remain unclear and the possibility of selection bias cannot be ruled out in the cross-sectional study. Other studies found a low participation of resettlers for other cancer detection programmes in Germany [28, 29]. Further research is needed to clarify the contradicting results. Therefore, the results from the NAKO study could be helpful.

## Data Availability

The datasets used and/or analysed during the current study are available from the corresponding author on reasonable request.

## Abbreviations

AD: Administrative district
AMIN study: Aussiedler in Münster - Incidence Cohort Study
ASRs: Age-standardised incidence rates
CI: Confidence interval
FSU: Former Soviet Union
InEMa study: Informierte Entscheidung zur Teilnahme am Mammographie-Screening-Programm
MSP: Mammography screening programme
NRW: North Rhine-Westphalia
OR: Odds ratio
UICC: Union for International Cancer Control

## References

[1] Gesellschaft der epidemiologischen Krebsregister in Deutschland. GEKID Atlas - Krebs gesamt (GEKID atlas - all cancer diagnoses) 2017. Available from: http://www.gekid.de/Atlas/CurrentVersion/atlas.html. Accessed 8 Mar 2018.

[2] Arnold M, Razum O, Coebergh JW (2010). Cancer risk diversity in non-western migrants to Europe: an overview of the literature. Eur J Cancer.

[3] John EM, Phipps AI, Davis A, Koo J (2005). Migration history, acculturation, and breast cancer risk in Hispanic women. Cancer Epidemiol Biomark Prev.

[4] Bulliard JL, De Landtsheer JP, Levi F (2004). Profile of women not attending in the Swiss mammography screening pilot Programme. Breast.

[5] Renshaw C, Jack RH, Dixon S, Møller H, Davies EA (2010). Estimating attendance for breast cancer screening in ethnic groups in London. BMC Public Health.

[6] Kristiansen M, Thorsted BL, Krasnik A, von Euler-Chelpin M (2012). Participation in mammography screening among migrants and non-migrants in Denmark. Acta Oncol.

[7] Martín-López R, Jiménez-García R, Lopez-de-Andres A, Hernández-Barrera V, Jiménez-Trujillo I, Gil-de-Miguel A (2013). Inequalities in uptake of breast cancer screening in Spain: analysis of a cross-sectional national survey. Public Health.

[8] Brown WM, Consedine NS, Magai C (2006). Time spent in the United States and breast cancer screening behaviors among ethnically diverse immigrant women: evidence for acculturation?. J Immigr Minor Health.

[9] Perry N, Broeders M, de Wolf C, Törnberg S, Holland R (2006). Karsa lv. European guidelines for quality assurance in breast cancer screening and diagnosis.

[10] Kaatsch P, Spix C, Katalinic A, Hentschel S, Luttmann S, Stegmaier C (2017). Krebs in Deutschland 2013/2014 (Cancer in Germany: 2013/2014).

[11] Giordano L, Von Karsa L, Tomatis M, Majek O, De Wolf C, Lancucki L (2012). Mammographic screening programmes in Europe: organization, coverage and participation. J Med Screen.

[12] Kooperationsgemeinschaft Mammographie (2015). Evaluationsbericht 2005–2012 - Ergebnis- und Prozessqualität im deutschen Mammographie-screening-Programm (evaluation report 2005–2012 - quality of the process and results of the German mammography screening program).

[13] Kooperationsgemeinschaft Mammographie (2016). Jahresbericht evaluation 2013 - Deutsches Mammographie-screening-Programm (annual evaluation report 2013 - German mammography screening program).

[14] Kooperationsgemeinschaft Mammographie (2016). Jahresbericht evaluation 2014 - Deutsches Mammographie-screening-Programm (annual evaluation report 2014 - German mammography screening program).

[15] Kooperationsgemeinschaft Mammographie (2017). Jahresbericht evaluation 2015 - Deutsches Mammographie-screening-Programm (annual evaluation report 2015 - German mammography screening program).

[16] Worbs S, Bund E, Kohls M (2013). Babka von Gostomski C. (Spät-) Aussiedler in Deutschland. Eine Analyse aktueller Daten und Forschungsergebnisse (Resettlers in Germany. An analysis of recent data and research findings).

[17] Statistisches Bundesamt (2019). Bevölkerung und Erwerbstätigkeit. Bevölkerung mit Migrationshintergrund. Ergebnisse des Mikrozensus 2018.

[18] Cho AB, Jaehn P, Holleczek B, Becher H, Winkler V (2018). Stage of cancer diagnoses among migrants from the former Soviet Union in comparison to the German population–are diagnoses among migrants delayed?. BMC Public Health.

[19] Kaucher S, Kajüter H, Becher H, Winkler V (2018). Cancer incidence and mortality among ethnic German migrants from the former Soviet Union. Front Oncol.

[20] Berens EM, Reder M, Kolip P, Spallek J (2014). A cross-sectional study on informed choice in the mammography screening programme in Germany (InEMa): a study protocol. BMJ Open.

[21] Marteau TM, Dormandy E, Michie S (2001). A measure of informed choice. Health Expect.

[22] Becher H, Winkler V (2017). Estimating the standardized incidence ratio (SIR) with incomplete follow-up data. BMC Med Res Methodol.

[23] Kajüter H, Batzler W, Krieg V, Heidinger O, Hense HW (2012). Abgleich von Sekundärdaten mit einem epidemiologischen Krebsregister auf der Basis verschlüsselter Personendaten–Ergebnisse einer Pilotstudie in Nordrhein-Westfalen (Linkage of Secondary Data with Cancer Registry Data on the Basis of Encrypted Personal Identifiers – Results from a Pilot Study in North Rhine-Westphalia). Das Gesundheitswesen.

[24] Krieg V, Hense HW, Lehnert M, Mattauch V (2001). Record Linkage mit kryptografierten Identitätsdaten in einem bevölkerungsbezogenen Krebsregister (Cryptographic Record Linkage in Population-based Cancer Registries). Das Gesundheitswesen.

[25] Gospodarowicz MK, Brierley JD, Wittekind C. TNM classification of malignant tumours. eighth Edition ed. Union for International Cancer Control (UICC), editor. Oxford, Hoboken: Wiley; 2017.

[26] Pace M, Lanzieri G, Glickman M, Zupanič T (2013). Revision of the European Standard Population: report of Eurostat's task force.

[27] Simbrich A, Wellmann I, Heidrich J, Heidinger O, Hense HW (2016). Trends in advanced breast cancer incidence rates after implementation of a mammography screening program in a German population. Cancer Epidemiol.

[28] Berens EM, Mohwinkel LM, van Eckert S, Reder M, Kolip P, Spallek J. Uptake of gynecological Cancer screening and performance of breast self-examination among 50-year-old migrant and non-migrant women in Germany: results of a cross-sectional study (InEMa). J Immigr Minor Health. 2018;21(3):1–4. 10.1007/s10903-018-0785-7 29987640

[29] Aparicio ML, Döring A, Mielck A, Holle R. Unterschiede zwischen Aussiedlern und der übrigen deutschen Bevölkerung bezüglich Gesundheit, Gesundheitsversorgung und Gesundheitsverhalten: eine vergleichende Analyse anhand des KORA-Surveys 2000 (Differences between Eastern European immigrants of German origin and the rest of the German population in health status, health care use and health behaviour: a comparative study using data from the KORA-Survey 2000) Soz Praventivmed. 2005;50(2):107–18. 10.1007/s00038-004-3088-9 15900963

[30] Kuhrs E, Winkler V, Becher H (2012). Risk factors for cardiovascular and cerebrovascular diseases among ethnic Germans from the former Soviet Union: results of a nested case-control study. BMC Public Health.

[31] Winkler V, Leitzmann M, Obi N, Ahrens W, Edinger T, Giani G (2014). Response in individuals with and without foreign background and application to the National Cohort in Germany: which factors have an effect?. Int J Public Health.

[32] Wichmann H-E, Kaaks R, Hoffmann W, Jöckel K-H, Greiser K, Linseisen J (2012). Die Nationale Kohorte. Bundesgesundheitsblatt Gesundheitsforschung Gesundheitsschutz.

